# The Story of the Insane from Year to Year

**Published:** 1906-09-08

**Authors:** 


### THE STORY OF THE INSANE FROM YEAR TO YEAR.

Eighteenth Annual Series.

### WILTS COUNTY ASYLUM.

On the first of January this asylum contained 977 patients (446 men and 531 women), and on the 31st of December there were 452 men and 536 women in residence, making a total of 988. There were 125 first admissions; 16 readmissions; 54 discharged recovered; 9 discharged relieved; and 66 died. The total number under treatment was 1,118, and the average daily number resident was 984. Calculated on the admissions, the recoveries were 38.3 per cent., and calculated on the average number resident the deaths were 6.7 per cent. Of the year's deaths 17 were caused by cerebral and spinal diseases; 5 by consumption, and 5 by other lung diseases. Of the year's admissions, 34 owed their insanity to such moral causes as domestic troubles, adverse circumstances, and religion; while among the physical causes hereditary influence heads the list with 46; previous attacks follow with 34, and drink comes next with 21. During the last five years this asylum, unlike most others, has experienced a fall in the number of admissions. In 1900 there were 189 admissions, and this number has been getting lower until 1905, when only 141 cases were admitted. In spite of this fact, the number of patients in the Asylum has risen during the same quinquennial period from 916 to 988; and Mr. Bowes shows that this increase is entirely owing to a decreasing death-rate which has dropped from 11 per cent. to 7.1 per cent. on the average number resident. Mr. Bowes adds, "it is improbable under the existing system of caring for and treating the insane that the death-rate will permanently revert to the high percentage of by-gone years; but as regards the admissions, there is less certainty." This is undoubted; but the tendency is almost invariably upwards, and in county asylums, at least, they would seem to rise in proportion, as wages are high and work plentiful. At any rate the Committee are unquestionably right in adding an additional fifty beds to the accommodation; and it would be most desirable to have a detached block for a like number of private patients, which would bring the total up to about 1,100 beds, beyond which it should under no circumstances be allowed to go. The Committee, "express their appreciation of the able manner in which, during the past, as in previous years, the Medical Superintendent has discharged the duties attached to his office." The weekly cost per head was 9s. 8⅝d. Private patients pay from 15s. to 21s.

### EAST RIDING ASYLUM, BEVERLEY.

This asylum contained 500 patients on the first of January, and on the thirty-first of December the number had risen to 546. There were 121 first admissions, 12 re-admissions, 30 discharged recovered, 4 discharged relieved, 13 discharged not improved, and 40 died. The total number under treatment during the year was 633, and the average daily number resident was 517. The recoveries were 31.57 per cent. of the admissions and the deaths were 7.73 per cent. of the average number resident, and both of these percentages are under the county asylum averages. Of the year's deaths, 11 were caused by cerebral and spinal diseases, 6 by consumption, 2 by other lung diseases, and 1 by enteric fever. Of the 133 admissions only 4 cases owed their insanity to moral causes; but in 24 instances the cause was hereditary influence; in 10 it was intemperance in drink,; and in 27 the cause was not ascertained. We have already remarked that the death-rate was lower than the average, and Dr. Macleod tells us "there has been an entire absence of cases of diarrhœa and dysentery"; but the asylum was not free from infectious disease, and he adds that there were seven cases of enteric fever, "the locality of the cases and the interval between their occurrence pointed to the absence of any general source of infection." The Commissioners in Lunacy remark that only 23 per cent. of the patients attend the weekly religious services, and they "observe that the chapel is far too small for the requirements of the asylum; and the question of increasing the accommodation should, we think, receive the early consideration of the Committee." If the Committee were to inquire what proportion of the 23 per cent. went of their free will, they would probably learn that the chapel was too large already. The Committee report that "the management of the asylum during the past year has been efficient in every department." The weekly charge for maintenance is 8s. 9d. per head for pauper patients, chargeable to the Riding. Out-county cases pay 14s. a week, and private cases from 14s. to 21s.

